# Improving primary care based post-diagnostic support for people living with dementia and carers: Developing a complex intervention using the Theory of Change

**DOI:** 10.1371/journal.pone.0283818

**Published:** 2023-05-03

**Authors:** Claire Bamford, Jane Wilcock, Greta Brunskill, Alison Wheatley, Karen Harrison Dening, Jill Manthorpe, Louise Allan, Sube Banerjee, Laura Booi, Sarah Griffiths, Greta Rait, Kate Walters, Louise Robinson, on behalf of the PriDem study team

**Affiliations:** 1 Population Health Sciences Institute, Faculty of Medical Sciences, Campus for Ageing and Vitality, Newcastle University, Newcastle upon Tyne, United Kingdom; 2 Research Department of Primary Care & Population Health, University College London, London, United Kingdom; 3 Voluntary Organisations Network North East, Newcastle, United Kingdom; 4 Dementia UK, London, United Kingdom; 5 NIHR Policy Research Unit in Health and Social Care Workforce, The Policy Institute at King’s, King’s College London, London, United Kingdom; 6 South Cloisters, University of Exeter, Exeter, United Kingdom; 7 Faculty of Health, University of Plymouth, Plymouth, United Kingdom; 8 Centre for Dementia Research, Leeds Beckett University, Leeds, United Kingdom; Indian Institute of Technology Jodhpur, INDIA

## Abstract

**Background:**

The need to improve support following a diagnosis of dementia is widely recognised, but it is unclear how this can best be achieved within UK health and social care systems. A task-shared and task-shifted approach has been recommended, but there is limited guidance on how to achieve this in practice. As part of a programme of research, we developed an intervention to enhance the role of primary care in post-diagnostic care and support for people living with dementia and carers.

**Methods:**

We used the Theory of Change to develop a complex intervention informed by initial literature reviews and qualitative work. The intervention was developed through an iterative series of workshops, meetings and task groups with a range of stakeholders, including the multidisciplinary project team, people living with dementia and carers, service managers, frontline practitioners, and commissioners.

**Results:**

142 participants contributed to intervention development through face-to-face or virtual meetings. The intervention comprises three complementary strands of work focusing on: developing systems, delivering tailored care and support, and building capacity and capability. Clinical dementia leads, based in primary care networks, will facilitate the intervention providing tailored expertise and support.

**Conclusion:**

The Theory of Change proved useful in providing structure and engaging stakeholders. The process was challenging, took longer and was less participative than intended due to restrictions caused by the COVID-19 pandemic. We will next conduct a feasibility and implementation study to explore whether the intervention can be successfully delivered within primary care. If successful, the intervention offers practical strategies for delivering a task-shared and task-shifted approach to post-diagnostic support that could be adapted for similar health and social care contexts internationally.

## Introduction

Globally there is growing emphasis on dementia as a policy priority, with countries developing national plans and guidelines [1, 2] to improve diagnostic rates [3], care [4, 5], and prevention [4]. Improving support to people living with dementia and their families following diagnosis is central to the Global Dementia Charter of 2013 [6]; and was identified as a key priority in a UK stakeholder exercise [7]. The emphasis on support is increasingly reflected in a policy focus on enabling people and families to live as well as possible with dementia [4, 8–11]. Current provision of care to people with dementia is, however, inadequate and inequitable both between and within countries [12, 13]. Specific populations have been shown to be under-served, including people from black and Asian minority ethnic groups [14, 15], those in rural and coastal areas [16, 17], and those with young onset dementia [18] and less common dementia subtypes such as dementia with Lewy bodies or frontotemporal dementia [19, 20].

A task-shared and task-shifted approach, involving ‘delegating selected tasks to existing or new health professional cadres with either less training or narrowly tailored training’ compared with traditional specialist secondary care services [12], has been recommended as a cost-effective approach to post-diagnostic support in the context of limited specialist resources and funding. This approach additionally seeks to develop the workforce and ensure that services are delivered by the most appropriate professionals [21, 22]. Greater involvement of primary and community care in post-diagnostic support has a number of other potential advantages including accessibility, and the potential to offer a more holistic approach, which is crucial given that almost 90% of people with dementia have at least one other long-term health condition [23, 24].

There is, however, limited evidence on the effectiveness of task-sharing and task-shifting and little guidance on implementation in practice either generally or specifically for dementia care. Existing literature focuses mainly on task-shifting *within* primary care (commonly nurses taking over tasks traditionally seen as within the medical domain [25]). Although management of some chronic diseases, for example, diabetes, asthma and heart failure, is now largely led by primary care in the UK, there is surprisingly little evidence to support task-sharing or task-shifting across sectors (e.g., from secondary care to primary care).

As part of the PriDem programme of research (https://research.ncl.ac.uk/pridem/) we explored how a task-shared and task-shifted approach to post-diagnostic support for people with dementia and carers could be operationalised in primary care. This paper summarises the process of intervention development and describes the resulting intervention.

## Methods

We aimed to develop an intervention to improve post-diagnostic dementia care and support underpinned by the following principles:

- Evidence-based and informed by good practice
- Led by primary care
- Person-centred, to meet current UK policy emphasis on personalised care [26]
- Sustainable.

### Design

We followed the Medical Research Council’s recommendations for complex intervention development, beginning with review and synthesis of evidence [27–36], then a mapping exercise [37] and detailed qualitative study of current provision [38–40]. Intervention development was informed by the principles of co-design, including valuing the different knowledge and experiences brought by stakeholders, striving to include marginalised groups, and enabling stakeholders to contribute in different ways [41].

**Fig 1:**
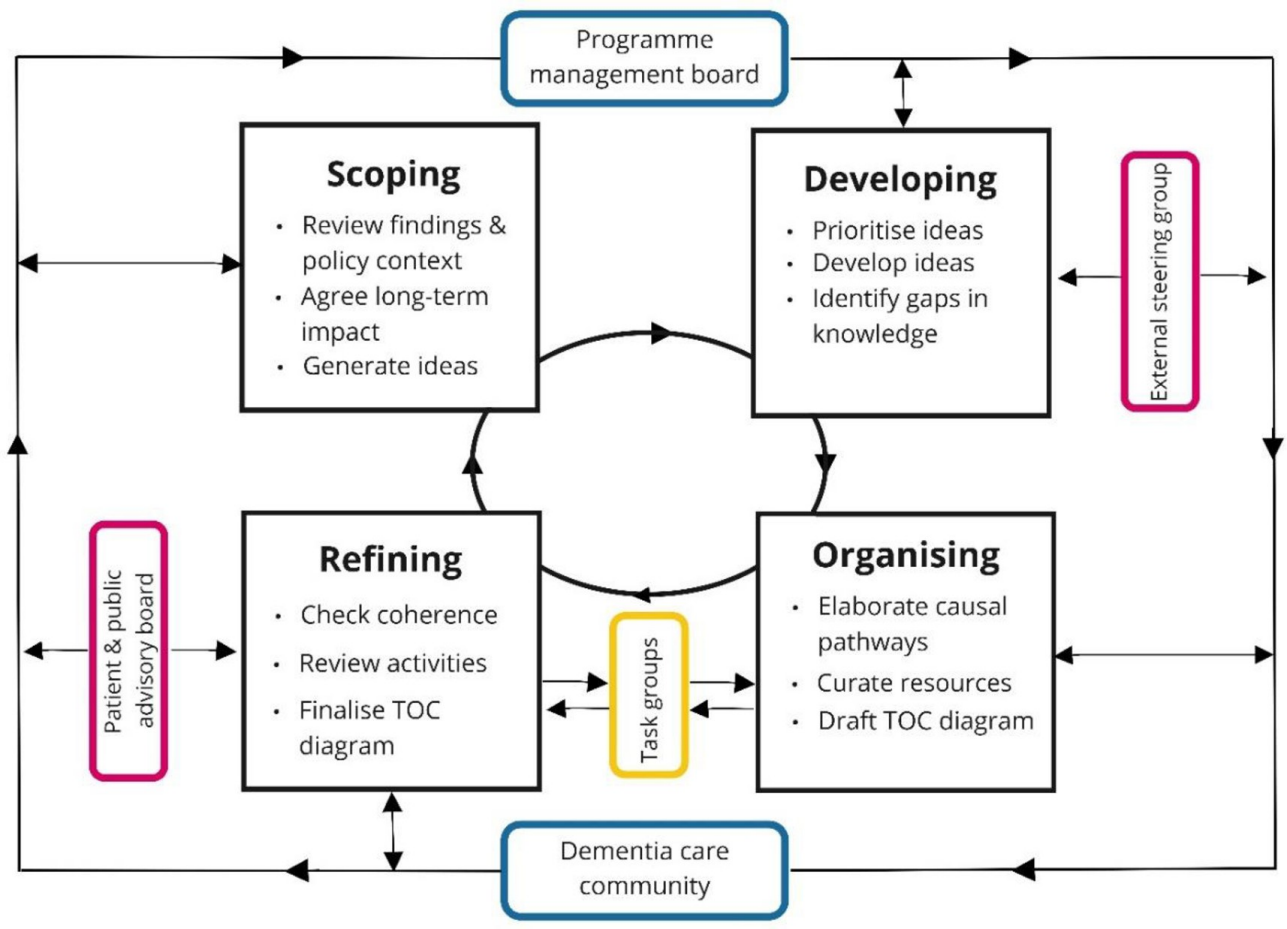
Process of developing the intervention.

We identified the Theory of Change (ToC) as an appropriate framework to systematically develop the intervention [42]. We define ToC as ‘a theory of how and why an initiative works which can be empirically tested by measuring indicators for every expected step on the hypothesised causal pathway to impact’ [42]. While the overall purpose of the ToC is well-established [42, 43], the process of developing a ToC varies [44, 45]. The process we followed is described below and summarised in Fig 1.

We supplemented the ToC with a detailed description of existing barriers to the provision of post-diagnostic dementia care [46], and identified appropriate methods to deliver intervention activities using evidence-based implementation strategies [47, 48] and behaviour change methods [49]. We followed recommendations to improve consistency of reporting of interventions [50, 51] and use of the ToC [42, 43] (see S1 File).

### Stakeholder involvement

Participating stakeholders are shown in Fig 1, with colours indicating the nature of their involvement.

- The Programme Management Board (PMB, 30 participants) included clinicians (general practitioners (GPs), old age psychiatrists, a geriatrician and a nurse with extensive experience of dementia care), methodologists, applied health care researchers with experience in dementia and two lay co-applicants with personal experience of caring for someone with dementia. PMB members contributed throughout the process of intervention development (indicated by a blue outline in Fig 1).
- The Dementia Care Community (DCC, 56 participants) comprised people living with dementia, informal carers (someone who provides unpaid help to a friend or family member, hereafter ‘carer’) and professionals involved in delivering dementia care from health, social care, third sector and private organisations. The DCC was established at the outset of the programme to provide ongoing advice on study conduct and review emerging findings. People with dementia and carers were recruited using a range of approaches including visits to local dementia cafes and groups and referrals by local professionals [52]. DCC members contributed throughout the process of intervention development (indicated by a blue outline in Fig 1).
- Feedback on the intervention was additionally obtained from the External Steering Committee (ESC, 10 participants) and the Public and Patient Advisory Board (PPAB, 8 participants). The ESC comprised academics and professionals with expertise in dementia, service provision, policy and implementation science. Their role was to provide expert advice independently of the investigators. The PPAB comprised carers with experience of dementia, largely recruited from Alzheimer’s Society Research Network carer groups, to provide comment and advice on the research programme. Both groups met once during the process of intervention development (indicated by a pink outline in Fig 1).
- Finally, we conducted a series of eight task groups and three individual interviews (38 participants in total). Participants included service managers, commissioners, frontline staff (from health, social care and the third sector), and people living with dementia and carers who had participated in earlier PriDem workstreams [37–40]. People with dementia and carers were recruited via service managers of six services selected as examples of innovative approaches to post diagnostic support [40]. Each participant contributed to a single task group, but ideas and comments from earlier task groups fed into subsequent groups (indicated by a yellow outline in Fig 1).

### Process of intervention development

Intervention development took place between September 2019 and May 2021. The development of the intervention involved scoping, developing, organising, and refining the intervention (Fig 1). These activities were iterative rather than a simple linear process. The first stage, scoping, comprised a series of iterative rapid appraisal workshops [53] with the PMB to review data, identify relevant policy, consider the overall aim of the intervention, and generate ideas to achieve this aim. The DCC similarly reviewed the findings from earlier programme workstreams and drew on their own experiences to identify key issues with current post-diagnostic support. This enabled us to agree the long-term intended impact of the intervention and start generating ideas.

During the developing stage we prioritised ideas according to feasibility and alignment with policy and developed selected ideas by considering what long-term outcomes would be required to achieve the desired impact. Following the principles of ToC, we then identified the preconditions (short- to medium-term outcomes) and how, where, and with whom the intervention should focus to achieve these long-term outcomes. During this process, gaps in our knowledge were identified and addressed through rapid syntheses of existing literature. The PMB, DCC and ESC reviewed these early ideas.

During the organising stage we identified activities to enable the preconditions to be met, sequenced these into causal pathways, and identified suitable implementation strategies or behaviour change techniques. We used an online whiteboard tool (https://miro.com) to develop a ToC diagram (visual overview of the intervention) to facilitate discussion during virtual interviews and presentations.

Finally, during the refining stage, the draft intervention was critically reviewed by a wide range of stakeholders through task groups and the PPAB for feedback on proposed activities and pathways, and additional ideas on putting the intervention into practice. The task groups spanned the organising and refining stages. After finalising the intervention, we produced a detailed narrative summary of the ToC and intervention manual (both available from https://doi.org/10.25405/data.ncl.c.5718116), and collated and developed resources required for implementation. A strapline (tailored, local, timely dementia support) and logo for the intervention were developed by the DCC using words identified as occurring frequently in the narrative summary of the ToC.

### Setting

Intervention development took place in England. Initial meetings of the PMB and DCC were face-to-face but subsequent meetings and task groups were conducted virtually due to the COVID-19 pandemic. People living with dementia and carers were offered a telephone call or practice video session to support participation. We adapted virtual DCC meetings by reducing the number of participants in each meeting but increasing opportunities for engagement of the wider DCC through feeding back key discussions for comment via individual virtual meetings, telephone calls, email and a bimonthly newsletter.

### Data collection

Meetings related to programme management (PMB, ESC, PPAB) were audio-recorded and summarised in detailed minutes, with key sections transcribed. DCC meetings with substantive discussion of aspects of the intervention were audio-recorded and relevant sections transcribed. PMB, DCC and ESC members additionally contributed through email, telephone calls and comments on documents. Task groups and interviews were audio-recorded and transcribed. All transcripts were checked and pseudonymised.

### Data analysis

Analysis took place in two phases. To maintain momentum, the initial emphasis was on rapidly synthesising key decisions, issues and questions from PMB and DCC discussions (JW, CB, LB, GB). This identified areas requiring clarification or elaboration and facilitated crossover of ideas and priorities between the PMB and DCC during the scoping and developing stages. Key comments and issues identified by the ESC, PPAB and task groups were similarly collated and fed back to the PMB and DCC.

In the second phase of analysis we used the framework approach to analyse transcribed data in more detail [54]. This approach is particularly appropriate for research improving effectiveness of systems and services [54], and allowed for integration of deductive and inductive codes, aiding capture of unanticipated, emerging issues such as the implications of COVID-19. We followed the five steps of framework analysis: familiarisation with the data; iteratively developing a framework or coding frame; indexing (or coding) transcripts; charting (producing tables summarising the views of stakeholder groups on each code); and finally mapping and interpreting, where we synthesised data after comparing discussions within and between stakeholder groups. We used NVivo 12 to assist with data management. An overview and description of key themes is provided in S2 File. We subsequently used the framework of context-mechanism-outcome configurations (CMOc) [55, 56] to clarify the hypothesised mechanisms through which we anticipated the intervention would achieve change.

### Ethics

Ethical approval was granted by NHS Research Ethics Committee Wales 3 (reference 18/WA/0349). PMB, DCC, ESC and PPAB members did not give formal written consent but provided verbal consent for audio recordings. Most task group participants provided formal written consent via email or post for study participation including audio recording, with a reminder to those from whom a completed consent form was not received. Verbal consent for audio-recording was confirmed at the start of all meetings and task groups.

## Results

### Participants

Participant characteristics are shown in Table 1. Researchers with a clinical background were coded according to their clinical role. Some participants contributed to multiple stages or multiple activities within a stage. The difference between total and unique participants demonstrates both continuity and new perspectives across stages. We collected limited demographic data, but include gender in Table 1.

**Table 1:** Stakeholders contributing to the ToC.

Stakeholder group	Stage of intervention development				Gender		Total participants	Total unique participants
	Scoping	Developing	Organising	Refining	M	F		
People living with dementia	17	3	3	6	10	12	29	22
Carers	15	8	11	33	12	27	67	39
Primary care	3	6	1	12	4	11	22	15
Secondary care	8	4	3	14	11	9	29	20
Social care	5	3	2	3	0	5	13	5
Third sector	6	6	3	9	1	13	24	14
Researchers	9	9	2	15	6	16	35	22
Cross sector				2	2	0	2	2
Policy leads	0	2	0	1	1	2	3	3
Total participants	63	41	25	95	47	95	224	142

We included people living with dementia, carers and professionals working with individuals from a range of marginalised groups, including Black, Asian and minority ethnic groups, those living in rural areas, and those with less common dementia subtypes such as young onset, Lewy body or frontotemporal dementia.

### Prioritising data from different sources

Since our intention was to develop a person-centred intervention, the voices of people living with dementia and carers were typically given precedence in decision-making. For example, there was a strong emphasis in PMB discussions on distinguishing between core and desirable components of post-diagnostic support. DCC members were therefore asked to review the components of post-diagnostic support identified through our qualitative work [40] to advise which were ‘core’. Members found this exercise challenging and strongly contested the proposed distinction between ‘core’ and ‘desirable’ components, arguing that all components were ‘core’ at the time they were needed and priorities were thought to be very personal:

*We thought that the needs that you have are different depending on the type of person you are or the severity of your illness, or your other medical problems. (Carer, DCC)*

As a result, this distinction was no longer seen as viable.

The literature review findings were not always consistent with other evidence. For example, while case management emerged as the most promising approach for enhancing post-diagnostic support in primary care [29], the relevance of case management to all people with dementia was contested by stakeholders who argued that while a named point of contact was important for all, the more intensive input associated with case management was only appropriate for people with complex needs. This lack of fit between the literature reviews and qualitative work reflects the focus of published studies on nurse-led case management, with limited exploration of alternative approaches. As a result, we adopted a broader approach which addressed systemic factors, rather than simply adding a case manager to the existing system [38].

### The PriDem intervention: Tailored, local, timely dementia support

The intended impact of the intervention is to enable people with dementia and carers to live as well as possible across the illness trajectory. The intervention focuses on supporting and developing the delivery of post-diagnostic support within primary care and will be delivered over a 12 month period. A summary of the intervention is provided below and a structured framework for describing interventions [51] is available in S1 File.

Overall views on the proposed intervention were generally positive, with stakeholders feeling confident that the intervention could make a difference:

*We talked about the struggles that we have had, and how difficult, [and] it’s actually quite reassuring to see that this work that’s being done is bringing something together that you can actually see would work. (Carer, DCC)*

**Fig 2:**
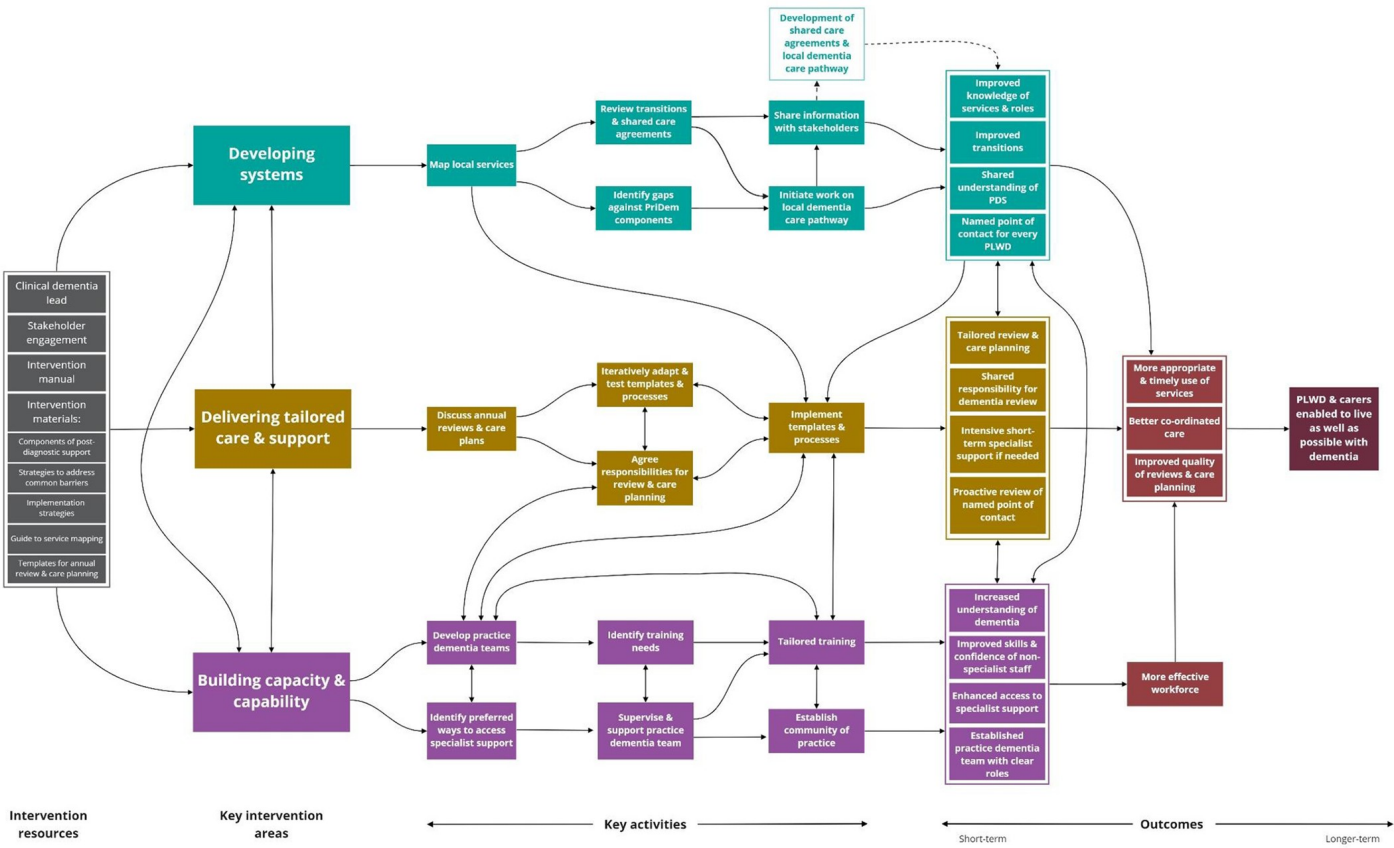
ToC diagram for the PriDem intervention.

The ToC diagram provides a visual representation of the intervention (Fig 2) including resources, and outcomes. When reviewing an early draft of the ToC diagram, PMB members commented that it was unclear how the intervention would benefit people living with dementia and carers. We therefore developed two fictional case studies to illustrate common shortcomings in existing post-diagnostic support and the improvements we anticipated the PriDem intervention would make to people living with dementia and carers (see [3]).

### Intervention content

The PriDem intervention includes three main strands:

- **Developing systems** for delivery of evidence-based, post-diagnostic support
- **Delivering tailored care and support** to meet the needs of with people with dementia and their carers
- **Building capacity and capability** by supporting non-specialists to deliver post-diagnostic dementia care.

Each intervention strand is described below. Activities relating to each intervention strand are summarised in the ToC diagram (Fig 2) and elaborated further in the intervention manual. Stakeholders considered the focus on three strands of work to be appropriate:

*I think you’ve managed to distil them, really well, into three really key areas and outline where the changes need to happen. (Clinical psychologist, interview)*

While we anticipate that emphasis on different intervention strands will vary according to local context, attention to all three is needed since there are strong interdependencies. For example, developing systems alone may result in limited changes if staff do not have the knowledge and skills to deliver tailored care. Similarly delivering tailored care is dependent on staff knowledge of local services, and skills and opportunities to provide such care. Focusing solely on upskilling staff would have limited impact if local systems do not support new ways of working or opportunities for ongoing review. Further details and key feedback on each intervention strand are summarised below.

Developing systems

A key barrier to effective post-diagnostic dementia support is fragile and fragmented services [38]. Service mapping, while challenging to keep up to date, was seen as a useful way of tackling this, with social prescribers (non-clinical staff who link patients in primary care with sources of support within the community to improve health and well-being [57]) identified as having a key role. Mapping was intended to facilitate task-sharing by increasing awareness of local resources, and ensure that the intervention complemented these rather than competing with them:

*…our narrative needs to include that you’re taking existing resources in order to do something better, and whatever, so this builds on what you’ve done rather than saying, “Get rid of all that, bring something in,” or, even worse, this needs to be angled in, but it doesn’t matter what else you’ve got around it. (Old age psychiatrist, PMB workshop)*

Transitions between services could be problematic for people living with dementia. While a lack of shared IT systems was identified as a key barrier to smooth transitions, this was agreed to be beyond the scope of the PriDem intervention. We have, however, sought to include activities which could ameliorate common issues with transitions, for example building on local initiatives for sharing information, and improving dissemination of care plans.

Synthesising mapping information into a care pathway was generally seen as beneficial, although the term ‘care pathway’ was contested by several stakeholders with some preferring ‘network’ which implied a less linear trajectory. The need to operationalise pathways in a person-centred way by tailoring inputs to individual needs and preferences was stressed by people living with dementia and carers.

Delivering tailored care and support

This strand centres on improving the annual dementia review for which GPs in England receive financial reimbursement [58]. Comments from task group and DCC members confirmed the inadequacies of the current approach in many practices:

*… we get a yearly review, but they never come and see my mum and dad. It’s just a general comment and they’re not considering my mum and dad’s individual needs. […] it’s the quality that’s the problem. (Carer, carer task group)*

Stakeholders made a range of practical suggestions to improve annual dementia reviews, for example by ensuring that other long-term conditions were reviewed alongside dementia, providing opportunities for the person living with dementia and carer to speak in private, and tailoring the approach to suit individual people living with dementia. Additional recommendations concerned the content of the review, for example, highlighting areas such as driving and oral care. Mindful of the limited capacity of GPs, exacerbated by the COVID-19 pandemic, there was support for the idea of sharing responsibility for annual dementia reviews between members of the primary care team.

Tailored care and support also involved ensuring that people living with dementia were under the care of appropriately skilled professionals:

*I think the idea of being able to have an extra person there if it is beyond the remit or capability of the person that you are used to seeing is a good thing. (Person with dementia, task group with people with dementia and carers)*

Based on their own experiences, task group participants suggested that this could be achieved through a step up/down model or liaison model (where more specialist professional(s) work alongside the existing provider, rather than care being transferred to a different individual). The continuity of a liaison model was valued by people living with dementia and carers. In the context of the intervention, using the clinical dementia lead (see intervention resources below) to provide additional support was seen as more feasible than introducing a step up/down approach:

*Because it’s so unpredictable with dementia. You can be going along fine one minute, and then something happens–and it doesn’t have to be a medical thing, it just as equally could be a social thing, and yet there’s a definite need–and you need to step in very quickly to support them, and then you can step back again. (GP commissioner, task group 5)*

Building capacity and capability

While a key component of building capacity was the provision of ‘training’, this was interpreted differently both within the project team and by task group members. For some participants, ‘training’ was interpreted as formal educational sessions with a desire to identify a programme in advance and define minimum training required for members of the practice dementia team:

*Quite rightly, the emphasis is on training. I suppose I wondered, do you have a sense of what that would look like, and where you’d be able to get that from? […] It’s going to need a really good, structured training programme behind it, and one that’s, I suppose, tailored to different needs and different audiences. That’s not going to be easy to find. (Clinical psychologist, interview)*

Others emphasised practical training tailored to individual needs and learning styles, and delivered through mechanisms such as mentoring, multidisciplinary team meetings (MDTs) or co-working. There was also recognition that developing confidence was as important as increasing knowledge. Access to the expertise of the clinical dementia lead through telephone advice and MDTs was a key way of providing ongoing support, de-escalating emerging problems, and avoiding unnecessary referrals to secondary care.

Developing practice dementia teams will also build capacity and capability, create impetus for the intervention, share responsibility for dementia care within the practice and enhance sustainability. Linking with existing practice and PCN structures such as frailty or care home teams was suggested as a way of engaging with practices and identifying potential members of practice dementia teams.

### Intervention resources

The key resource to be introduced is a clinical dementia lead (CDL) to facilitate the intervention. The CDL will have dementia expertise, leadership experience and the ability to work with stakeholders to influence practice. Following initial training in the intervention, they will be provided with clinical supervision and intervention supervision (to provide support with aspects of the intervention that prove challenging, identify additional CDL training needs, and to identify any necessary modifications to the intervention). The need for appropriate supervision was highlighted by several task group participants:

*make sure you know who’s going to be their supervisors, because they need support. That has been a massive gap for us because, if you want the experts, they have to be supported. It’s not just good enough to get them in post. (Community dementia practitioner, professional task group 1)*

While some stakeholders drew parallels with other roles such as Admiral Nurses or Community Psychiatric/Mental Health Nurses linked to individual practices, these professionals typically focus primarily on providing direct care to people living with dementia and carers. The CDLs will explicitly work at a systems level with local providers and practices. Their role is therefore that of a catalyst for multi-level change as well as supporting primary care teams, and having a small caseload of people with dementia and carers with complex needs.

In addition to dementia expertise, participants stressed the value of experience in both physical and mental health. While professionals with a nursing background and specialist dementia knowledge were often seen as most relevant to the CDL role, occupational therapists [OTs] were identified as an appropriate alternative:

*The group that springs to mind for me is OTs because, in their basic training, they cover mental health as well as physical, so they’ve got quite a unique training, actually. It’s very person-centred right from the start, and those that go on to specialise in care of older people invariably know a lot about dementia. (Specialist dementia nurse consultant, professional task group 4)*

Materials to support CDLs in their role include a detailed intervention manual containing: activities and implementation strategies relating to each strand of the intervention; details of components of post-diagnostic support [40]; a guide to mapping services; and templates for annual dementia review and care planning. A training programme for CDLs has been developed and resources to build capacity and capability of primary care staff have been collated for use by the CDLs.

### Location in primary care networks

The intervention will take place across primary care networks (PCNs) which are organisational structures of between 1–10 practices serving between 30,000 and 50,000 patients. PCNs aim to: introduce new staff roles (e.g. pharmacist, paramedic); take a proactive approach to the wider health of their population; and work with local providers to deliver personalised, coordinated, health and care support [26]. There was strong support from most stakeholders for basing the intervention in PCNs:

*I think you probably will find people who are working in a PCN, the clinical directors and so forth, are actually also a bit motivated to look at something new and look at change. I think there probably is a bit of an appetite for them to try something new and support something new […] it would feel like a good fit. (GP, professional task group 1)*

Questions were raised about the number of CDLs that would be required to effect the changes needed in a PCN. Workload, demand and delivery will be assessed in the feasibility and implementation study. Basing the post at PCN level rather than within individual practices was proposed partly to promote sustainability, but also to try to avoid creating dependency on the CDL, as some task group participants had directly experienced problems when a practice-based service was withdrawn:

*I never really gave any consideration which just shows the quality of work that she [Admiral Nurse] was doing. And she was always there, that you could contact her anytime. It was such a support. And when she left it was such a black hole. (Carer, carer task group)*

Potential disadvantages of PCNs were the possibility of duplicating existing posts and difficulties in engagement if dementia was not a priority area for the PCN. There was acknowledgement that primary care would not be able to meet the needs of everyone with dementia and would require rapid access to specialist support; such services will be identified through the mapping exercise.

### Intervention delivery

Support and training for professionals will be delivered either individually or in groups through meetings and workshops, MDTs and case discussions, telephone and email advice, mentoring and co-working, with more formal training if required. Activities will be tailored according to the engagement of practice staff, preferred learning approaches, willingness to review and develop their existing annual dementia review template, and success in establishing a practice dementia team.

Direct support to people with dementia and carers will be provided through face to face consultations, home visits, telephone and email and will be tailored to their individual needs, existing support mechanisms and circumstances.

### Intervention fidelity

Deliverables and outputs relating to each intervention activity are documented in the intervention manual and will provide a framework for assessing fidelity through regular review in intervention supervision sessions with CDLs. Intervention supervision will be provided by senior researchers and a specialist dementia nurse consultant to review progress and trouble-shoot problems. Sessions will be fortnightly initially, then reduce in frequency as appropriate.

The use of the care planning template will be examined by audits before and after the introduction of the intervention. Engagement of individual practices and practitioners will be reviewed, including a log of mentoring, informal and formal training.

### Elaboration of mechanisms

While the arrows in the ToC diagram indicate the pathways through which we anticipate changes will occur, we have elaborated the hypothesised mechanisms using context-mechanism-outcome configurations [55, 56] (Table 2).

**Table 2:** Proposed mechanisms of change for the PriDem intervention.

Context	Mechanism (resource) *The intervention component added*	Mechanism (reasoning) *The change in psychology or behaviour produced by the resource*	Outcome
**CMOc1**			
Fragile & fragmented services mean that professionals may be unaware of local services available or how to access them leading to a sense that ‘nothing can be done’	• Mapping local services through joint work with stakeholders & sharing information with practice staff	• Increased awareness of local services • Improved understanding of roles & responsibilities • Reduction in therapeutic nihilism	• People living with dementia (PLWD) & carers are referred promptly & appropriately
**CMOc2**			
Commissioners receive limited guidance on how to negotiate local service provision	• Comparison of local services with NICE recommended interventions & PriDem components of post-diagnostic support	• Increased awareness of gaps & duplication in local provision	• Commissioners have evidence to inform future commissioning decisions
**CMOc3**			
Lack of joined up services & shared information systems create delays & duplication, & do not support the delivery of personalised care	• Review of transitions & information sharing	• Improved understanding of how & when to refer PLWD & carers • Timely provision of/access to relevant information	• Better co-ordinated care for PLWD & carers
**CMOc4**			
Non-specialists receive limited training on dementia & may lack knowledge, skills & confidence	• Provide tailored training & supervision based on experiential learning	• Improved knowledge, skills & confidence of non-specialists	• PLWD & carers receive appropriate & timely interventions & support
**CMOc5**			
Difficulties in accessing specialist advice on dementia for non-specialists	• Review shared care agreements • A Clinical Dementia Lead is available, as part of the intervention, to provide support & advice to non-specialists as well as direct care to people with complex needs where required	• Non-specialists understand their responsibilities, feel supported & can access advice	• Non-specialists are better equipped to deliver post-diagnostic dementia care & support • PLWD with complex needs can receive short-term intensive support from the CDL
**CMOc6**			
Variable quality of QOF annual dementia review & care planning undermines the delivery of personalised care	• Develop & implement templates & processes to improve review & care planning including sharing of care plans with PLWD, carers & other agencies	• Practice staff follow a clear process & agreed templates for review & care planning • Practice staff share care plans with other providers	• Needs are identified & appropriate support provided promptly • PLWD & carers are better informed about their care • Enhanced coordination of care
**CMOc7**			
Arrangements for providing & reviewing a suitable named point of contact may be unclear	• Robust arrangements for allocating & reviewing a named point of contact, linked to annual dementia review • Clear criteria for short-term specialist input from the CDL when needed	• PLWD, carers & professionals know who to contact about their care • PLWD & carers feel more confident about asking for help as they know support will be available	• PLWD are supported by someone whose skills & knowledge match their needs • Enhanced co-ordination of care
**CMOc8**			
Dementia leads in primary care may be isolated & reliance on a single individual may create problems with sustainability	• Create a practice dementia team • Establish a community of practice	• Practice staff create momentum for change • Workload is shared across the practice dementia team • Practice staff share experiences across sites & learn from one another	• Sustainable forums for ongoing development

### Assumptions underlying the ToC

Assumptions relate to areas of the ToC where the evidence is ‘weak, untested or uncertain’ [45]. Critical review of the intervention by internal and external stakeholders identified implicit assumptions relating to delivery, impact and process, and potential unintended consequences [45]. Key assumptions relate to capacity and willingness of stakeholders to engage with the intervention, including:

- a full-time CDL will be sufficient resource to support two PCNs
- primary care staff will be willing and have capacity to engage with the intervention
- people living with dementia and carers will be interested in engaging with the intervention, and
- the CDLs will successfully deliver all three intervention strands.

For each assumption, we collated supporting and refuting evidence drawn from the literature and/or prior experience of the research team. Identification of the assumptions was key to informing some of the processes, notably the supervision arrangements for the CDLs to support them in their new role.

### Tensions and uncertainties

During the process of intervention development there were tensions and uncertainties over specific aspects of the intervention. For example, within the PMB implicit assumptions regarding the scope of the intervention were not acknowledged leading to tensions between the desire to develop a multi-faceted, complex intervention and a preference for a smaller scale, less complex intervention. Other tensions related to the nature of the training to be offered/required of participating GP practices and whether they should receive funding to facilitate their involvement. Many of these tensions could not be resolved in the development phase but will instead be explored during the implementation of the intervention.

### Key changes during the process of intervention development

Initial ideas about the intervention focused on providing practical support for commissioners to ensure that all components of post-diagnostic support (including NICE recommended interventions [5]) were available locally. This idea was supported by the absence of user-friendly guidance for commissioners, evidence of a lack of support for their role [59], and qualitative work highlighting the key role of commissioners in good practice sites. However, the ESC strongly advised against a focus on commissioners due to: changes in their role resulting from the COVID-19 pandemic; the incompatibility of commissioning timescales with a relatively short-term intervention; and the limited success of previous interventions with commissioners [60]. This led to a decision that targeting the intervention at commissioners was not feasible. Since aspects of the final intervention will be of value to commissioners, we will share relevant resources and information to inform future commissioning cycles.

One proposed initiative to improve information sharing and facilitate transitions was the introduction of a patient-held record, to be maintained by the person with dementia and/or carer and available to professionals. This received a mixed response from task group and DCC participants, with reservations expressed about:

- Practicality (e.g., record being misplaced or not brought to appointments)
- Whether and how well professionals would use the record
- Additional responsibility for people living with dementia/carers to ensure the record was maintained, particularly during the COVID-19 pandemic where face-to-face contact was minimal.

There was also a lack of consensus over the content and format of a patient-held record. While there was a general view that it would need to be simple, this was inconsistent with discussions over what it should include. Given the absence of clear support for, and scant evidence of, the use of patient-held records in dementia, we decided against this approach.

## Discussion

This paper describes the development and content of a complex intervention which seeks to improve post-diagnostic support to enable people to live as well as possible with dementia. This is a key priority for people living with dementia [6, 7] and policy makers [9, 12] but achieving this within overstretched care systems is challenging. Here we operationalise a task-shared and task-shifted approach [12], designed for UK systems, providing explicit guidance on how to achieve this in practice.

Many previous interventions to improve primary care dementia management have focused on a single activity in isolation (e.g. training staff [61]) or have targeted a specific aspect of post-diagnostic support, (e.g. advance care planning [62] or end of life care [35]). Our detailed problem analysis [46] informed the content of the intervention by highlighting three key factors influencing the provision of post-diagnostic dementia care and support: (i) lack of a supportive infrastructure; (ii) limited proactive review; and (iii) limited capacity and capability particularly in primary care [38]. Our PriDem intervention aims to address these factors. While a multi-faceted complex intervention is inevitably more challenging to deliver, the need to focus on system level changes in developing interventions to improve dementia care is clear [63–65].

Our experience highlights the challenges of setting intervention parameters at the funding stage before the scope of the problem is fully explored. Working at systems level had not been anticipated, raising concerns over whether the funding allocated for the professionals delivering the intervention was sufficient. Similar problems have been reported elsewhere [66] suggesting that greater flexibility over the extent to which interventions are pre-specified and costed may be needed to ensure that interventions are able to address key barriers without being unduly constrained by limited funding.

The COVID-19 pandemic has created new pressures on primary care [67–69], which required us to consider how the intervention would align with reduced capacity within primary care and the shift to virtual consultations. This was partly addressed through the emphasis on task-sharing within the primary care team. The growing evidence that people living with dementia and carers have been amongst disproportionately affected by the COVID-19 pandemic [70–72] may also increase professional engagement with the intervention. Further, the COVID-19 pandemic has supported new ways of working, particularly virtual meetings, which may facilitate aspects of the intervention (e.g., MDT meetings) [73, 74].

The ToC proved useful in thinking through the sequence of events needed to achieve the desired end point. While not included in a checklist for reporting intervention development [50], we found the process of identifying assumptions, together with confirming and refuting evidence useful in making assumptions explicit and recommend actively including this step of the ToC in intervention development. Supplementing the ToC with a problem logic model (drawn from intervention mapping [46]) helped to conceptualise key barriers and ensured that the proposed intervention directly addressed these. The broader behaviour change and implementation literature [47, 48, 75] enabled us to identify appropriate strategies for key intervention activities. We also found it useful to elaborate the proposed mechanisms of change using context-mechanism-outcome configurations [55, 56].

### Strengths and limitations

An important strength of this work is the high level of meaningful involvement with professionals, people living with dementia and carers that we achieved, even during the COVID-19 pandemic. The majority of DCC members became involved when the group was formed at the outset of the programme and consequently had an in-depth understanding of the project as a whole and an established relationship with facilitators, enabling them to challenge aspects of the intervention they viewed as inappropriate or impractical.

Extensive pre-specified literature reviews were conducted at the outset, with limited resources for further focused reviews to inform specific aspects of the proposed intervention (e.g., how dementia reviews are currently performed; how best to develop capacity and capability in primary care).

Intervention development took longer than anticipated, largely due to the COVID-19 pandemic, which required us to pause for several months and then shift to virtual events rather than the planned face-to-face, more interactive and creative workshops.

## Conclusions

The PriDem intervention provides an evidence-based approach to post-diagnostic support for people living with dementia and carers. The feasibility and implementation of the intervention will now be tested across several primary care networks in England. The activities and change mechanisms proposed in the ToC will inform data collection and contribute to the assessment of intervention fidelity. If successful, the intervention offers strategies for a task-shared and task-shifted approach that could be adapted for similar health and social care contexts internationally.

## Supporting information

S1 File Reporting checklists. GUIDED–a guideline for reporting for intervention development studies; Checklist for reporting Theory of Change in Public Health Interventions; Intervention description and replication (TIDieR) checklist.
(DOCX)

S2 File Overview of key analytic themes. (DOCX)

S3 File Illustrative case studies. (DOCX)
