# Supplementary material for: Improving primary care based post-diagnostic support for people living with dementia and carers: Developing a complex intervention using the Theory of Change
Source: PLoS One. 2023 May 3;18(5):e0283818. doi: 10.1371/journal.pone.0283818 (PMC10155958; doi:10.1371/journal.pone.0283818)
Supplement: S1 File — GUIDED–a guideline for reporting for intervention development studies; Checklist for reporting Theory of Change in Public Health Interventions; Intervention description and replication (TIDieR) checklist. (DOCX) [file pone.0283818.s001.docx]

## Supplementary file 1: Reporting guidelines and checklists

- Checklist for reporting Theory of Change in Public Health Interventions
- GUIDED – a guideline for reporting for intervention development studies
- Description of the PriDem intervention using the TIDieR framework

**Checklist for reporting Theory of Change in Public Health Interventions** [1]

|  |  | Page number |
| --- | --- | --- |
| 1. | Is the ToC approach defined? |  |
| 1.1 | Is a definition of ToC given by the authors? | 5 |
| 1.2 | Do the authors explain their reasons for using a ToC approach? | 5, 27 |
| 2. | Is the ToC development process described? |  |
| 2.1 | Are the methods used to develop the ToC, such as stakeholder meetings and interviews, document reviews, programme observation, existing conceptual frameworks or published research, described? | 5-8, Figure 1 |
| 2.2 | Where stakeholders are involved, is it clear how many stakeholders participated, what their role is in relation to the intervention, how they were consulted (e.g. number of interviews, focus groups, ToC workshops) and the extent to which the consultations were participatory? | 6-8, 11-12, Table 1 |
| 2.3 | Is the method used to compile the data into a ToC described? (including how disagreements between stakeholders were resolved) | 6-8, 11-12, Figure 1 |
| 2.4 | Is the extent to which stakeholders were able to validate the resultant ToC and were owners of the final product described? | 7-8, 25-26, 28 |
| 3 | Is the resultant ToC (or a summary thereof) depicted in a diagrammatic form and does it include? |  |
| 3.1 | The long-term outcome or impact of the intervention | Figure 2 |
| 3.2 | The anticipated short- and medium-term outcomes and the process of change | 20-25, Figure 2, Table 2 |
| 3.3 | The intervention components which happen at different stages of the pathway | Figure 2, intervention manual |
| 3.4 | The context of the intervention | 3-4, 13-19, 27 |
| 3.5 | Assumptions about how change would occur | 20-25, Figure 2, Table 2 |
| 3.6 | Additional ToC elements such as indicators, supporting research evidence, beneficiaries, actors in the context, sphere of influence and timelines where relevant. | Intervention manual, detailed narrative summary of the ToC |
| 4. | Is the process of intervention development from the ToC described? |  |
| 4.1 | Are the methods of how interventions were refined from the ToC to something which can be implemented described? (For example, further stakeholder workshops, interviews, systematic literature reviews) | 11-26 |
| 5. | Is the way in which the ToC was used to develop and implement the evaluation described? | This section is of limited relevance in this paper which focuses on intervention development. |
| 5.1 | Are evaluation research questions generated from the ToC? | NA |
| 5.2 | Is the role of ToC in the design, plan or conduct of the evaluation clear? | An explanation of how the ToC will contribute to intervention supervision is provided on page |
| 5.3 | Does the paper describe the extent to which the key elements described in the ToC were measured in the evaluation (i.e. impact, short- and medium-term outcomes and the process of change, context, assumptions and the intervention)? | NA |
| 5.4 | Does the paper describe whether and how process indicators were used to improve the quality of the intervention? | An explanation of how indicators will be used in intervention supervision is provided on page |
| 5.5 | Is the role of the ToC in the analysis of the results of the evaluation clear? | NA |
| 5.6 | Is the role of ToC in the interpretation of the results of the evaluation described? (including the breakdown of programme theory, unanticipated outcomes and causation including the strength and direction of causal relationships) | NA |

**GUIDED – a guideline for reporting for intervention development studies** [2]

| **Item description** | | **Explanation** | **Page in manuscript where item is located** | **Other*** |
| --- | --- | --- | --- | --- |
| 1. | Report the context for which the intervention was developed. | Understanding the context in which an intervention was developed informs readers about the suitability and transferability of the intervention to the context in which they are considering evaluating, adapting or using the intervention. Context here can include place, organisational and wider socio-political factors that may influence the development and/or delivery of the intervention. | 3-4, 13-19, 27 |  |
| 2. | Report the purpose of the intervention development process. | Clearly describing the purpose of the intervention specifies what it sets out to achieve. The purpose may be informed by research priorities, for example those identified in systematic reviews, evidence gaps set out in practice guidance such as The National Institute for Health and Care Excellence or specific prioritisation exercises such as those undertaken with patients and practitioners through the James Lind Alliance. | 3-5 |  |
| 3. | Report the target population for the intervention development process. | The target population is the population that will potentially benefit from the intervention – this may include patients, clinicians, and/or members of the public. If the target population is clearly described then readers will be able to understand the relevance of the intervention to their own research or practice. Health inequalities, gender and ethnicity are features of the target population that may be relevant to intervention development processes. | 12-19 | Supplementary file 3 |
| 4. | Report how any published intervention development approach contributed to the development process | Many formal intervention development approaches exist and are used to guide the intervention development process (e.g. 6Squid or The Person Based Approach to Intervention Development). Where a formal intervention development approach is used, it is helpful to describe the process that was followed, including any deviations. More general approaches to intervention development also exist and have been categorised as follows:- Target Population-centred intervention development; evidence and theory-based intervention development; partnership intervention development; implementation-based intervention development; efficacy-based intervention development; step or phased-based intervention development; and intervention-specific intervention development. These approaches do not always have specific guidance that describe their use. Nevertheless, it is helpful to give a rich description of how any published approach was operationalised | 5-6 |  |
| 5. | Report how evidence from different sources informed the intervention development process. | Intervention development is often based on published evidence and/or primary data that has been collected to inform the intervention development process. It is useful to describe and reference all forms of evidence and data that have informed the development of the intervention because evidence bases can change rapidly, and to explain the manner in which the evidence and/or data was used. Understanding what evidence was and was not available at the time of intervention development can help readers to assess transferability to their current situation. | Figure 1. 5-9, 11-12, 25-26 |  |
| 6. | Report how/if published theory informed the intervention development process. | Reporting whether and how theory informed the intervention development process aids the reader’s understanding of the theoretical rationale that underpins the intervention. Though not mentioned in the e-Delphi or consensus meeting, it became increasingly apparent through the development of our guidance that this theory item could relate to either existing published theory or programme theory. | 5-6 |  |
| 7. | Report any use of components from an existing intervention in the current intervention development process. | Some interventions are developed with components that have been adopted from existing interventions. Clearly identifying components that have been adopted or adapted and acknowledging their original source helps the reader to understand and distinguish between the novel and adopted components of the new intervention. | NA |  |
| 8. | Report any guiding principles, people or factors that were prioritised when making decisions during the intervention development process. | Reporting any guiding principles that governed the development of the application helps the reader to understand the authors’ reasoning behind the decisions that were made. These could include the examples of particular populations who views are being considered when designing the intervention, the modality that is viewed as being most appropriate, design features considered important for the target population, or the potential for the intervention to be scaled up. | 5, 11-12, 25-26 |  |
| 9. | Report how stakeholders contributed to the intervention development process. | Potential stakeholders can include patient and community representatives, local and national policy makers, health care providers and those paying for or commissioning health care. Each of these groups may influence the intervention development process in different ways. Specifying how differing groups of stakeholders contributed to the intervention development process helps the reader to understand how stakeholders were involved and the degree of influence they had on the overall process. Further detail on how to integrate stakeholder contributions within intervention reporting are available. | 5-26, Figure 1 |  |
| 10. | Report how the intervention changed in content and format from the start of the intervention development process. | Intervention development is frequently an iterative process. The conclusion of the initial phase of intervention development does not necessarily mean that all uncertainties have been addressed. It is helpful to list remaining uncertainties such as the intervention intensity, mode of delivery, materials, procedures, or type of location that the intervention is most suitable for. This can guide other researchers to potential future areas of research and practitioners about uncertainties relevant to their healthcare context. | 11-12, 25-26 |  |
| 11. | Report any changes to interventions required or likely to be required for subgroups. | Specifying any changes that the intervention development team perceive are required for the intervention to be delivered or tailored to specific sub groups enables readers to understand the applicability of the intervention to their target population or context. These changes could include changes to personnel delivering the intervention, to the content of the intervention, or to the mode of delivery of the intervention. | 14-16 |  |
| 12. | Report important uncertainties at the end of the intervention development process. | Intervention development is frequently an iterative process. The conclusion of the initial phase of intervention development does not necessarily mean that all uncertainties have been addressed. It is helpful to list remaining uncertainties such as the intervention intensity, mode of delivery, materials, procedures, or type of location that the intervention is most suitable for. This can guide other researchers to potential future areas of research and practitioners about uncertainties relevant to their healthcare context. | 24-25 |  |
| 13. | Follow TIDieR guidance when describing the developed intervention. | Interventions have been poorly reported for a number of years. In response to this, internationally recognized guidance has been published to support the high quality reporting of health care interventions and public health interventions. This guidance should therefore be followed when describing a developed intervention | Included in S1 file | See next table |
| 14. | Report the intervention development process in an open access format. | Unless reports of intervention development are available people considering using an intervention cannot understand the process that was undertaken and make a judgement about its appropriateness to their context. It also limits cumulative learning about intervention development methodology and observed consequences at later evaluation, translation and implementation stages. Reporting intervention development in an open access (Gold or Green) publishing format increases the accessibility and visibility of intervention development research and makes it more likely to be read and used. Potential platforms for open access publication of intervention development include open access journal publications, freely accessible funder reports or a study web-page that details the intervention development process | To be reported in open access journal |  |

*e.g. if item is reported elsewhere, then the location of this information can be stated here.

**Description of the PriDem intervention using the TIDieR framework** [3]

| **BRIEF NAME** | | **Page number** | **Line number** |
| --- | --- | --- | --- |
| **1.** | Provide the name or a phrase that describes the intervention. | 12 | 251 |
| **WHY** | |  |  |
| **2.** | Describe any rationale, theory, or goal of the elements essential to the intervention. | 3-4  13  20-24 | 53-83  270-275  Table 2 |
| **WHAT** | |  |  |
| **3.** | Materials: Describe any physical or informational materials used in the intervention, including those provided to participants or used in intervention delivery or in training of intervention providers. Provide information on where the materials can be accessed (e.g., online appendix, URL). | 8, 13  Figure 2  Detailed narrative summary of the ToC  Intervention manual | 170-173  278  <https://doi.org/10.25405/data.ncl.c.5718116>  <https://doi.org/10.25405/data.ncl.c.5718116> |
| **4.** | Procedures: Describe each of the procedures, activities, and/or processes used in the intervention, including any enabling or support activities. | 12-16  Intervention manual | 269-358  <https://doi.org/10.25405/data.ncl.c.5718116> |
| **WHO PROVIDED** | |  |  |
| **5.** | For each category of intervention provider (e.g., psychologist, nursing assistant), describe their expertise, background and any specific training given. | 16-18 | 359-390 |
| **HOW** | |  |  |
| **6.** | Mode of delivery | 19 | 417-425 |
| **WHERE** | |  |  |
| **7.** | Location(s) where the intervention will occur, including any necessary infrastructure or relevant features. | 18-19 | 391-416 |
| **WHEN and HOW MUCH** | |  |  |
| **8.** | The number of times the intervention will be delivered and over what period of time. | 19 | 417-425 |
| **TAILORING** | |  |  |
| **9.** | If the intervention is planned to be personalised, titrated or adapted, then describe what, why, when, and how. | 19 | 417-425 |
| **MODIFICATIONS** | |  |  |
| **10.** | If the intervention was modified during the course of the study, describe the changes (what, why, when, and how). | N/A |  |
| **HOW WELL** | |  |  |
| **11.** | Planned: If intervention adherence or fidelity will be assessed, describe how and by whom, and if any strategies will be used to maintain or improve fidelity, describe them. | 19 | 426-434 |
| **12.** | Actual: If intervention adherence or fidelity was assessed, describe the extent to which the intervention was delivered as planned. | N/A |  |

**References**

1. Breuer E, Lee L, De Silva M, Lund C. Using theory of change to design and evaluate public health interventions: a systematic review. Implementation Science. 2016;11(63):1-17. doi: <https://doi.org/10.1186/s13012-016-0422-6>.

2. Duncan E, Cathain A, Rousseau N, Croot L, Sworn K, Turner KM, et al. Guidance for reporting intervention development studies in health research (GUIDED): an evidence-based consensus study. BMJ Open. 2020;10(4):e033516. doi: 10.1136/bmjopen-2019-033516.

3. Hoffman TC. Better reporting of interventions: a template for intervention description and replication (TIDieR) checklist and guide. BMJ. 2014;348(g1687). doi: <https://doi.org/10.1136/bmj.g1687>.
