# Supplementary material for: Improving primary care based post-diagnostic support for people living with dementia and carers: Developing a complex intervention using the Theory of Change
Source: PLoS One. 2023 May 3;18(5):e0283818. doi: 10.1371/journal.pone.0283818 (PMC10155958; doi:10.1371/journal.pone.0283818)
Supplement: S2 File — (DOCX) [file pone.0283818.s002.docx]

## **Overview of themes**

##

| **Theme** | **Description** |
| --- | --- |
| Fit of review & qualitative findings with clinical & practice experience | Whether the findings contained any surprises or omissions  Nature of ‘evidence’ |
| Defining the scope of the intervention | Identifying the scope & ultimate aim of the intervention  Whether intervention components could & should be prioritised  Identifying the target audience of the intervention (e.g. which people with dementia & carer should have access to the intervention? Which members of the primary care team should be involved?) |
| Fit with policy initiatives | Alignment of the intervention with current policy  Ability of the intervention to adapt to changing service landscapes & priorities |
| Costs & sustainability | Population that could feasibly be covered by a single CDL  Perceived willingness of commissioners & primary care staff to invest in the intervention |
| Gaps in our knowledge | Identification of areas where more information was needed (e.g. patient held records) |
| Who should deliver the intervention? | Professional background(s) perceived as most relevant for the CDLs |
| Location of the intervention | Advantages & disadvantages of basing the intervention in Primary Care Networks |
| Engaging stakeholders in the intervention | How to obtain buy-in to the intervention in the context of competing priorities |
| Tailoring the intervention to the local context | Understanding the local context (through service mapping)  Building on & integrating with existing initiatives |
| Role of CDL in providing direct support to people with dementia & carers | Protecting time for developing systems & upskilling staff  Fit of CDL with existing services & options for working as a step up/down model |
| Developing annual review & care planning | Scope for developing review & care planning  Examples of existing good practice |
| Feasibility & acceptability of introducing a patient held record | Extent to which successfully embedded in other conditions  Perceived relevance to dementia |
| Developing capacity & capability | Identifying members of the primary care team for upskilling  Appropriate approaches to upskilling |
| Viability of the intervention in a [post] COVID-19 landscape | Impacts of COVID-19 on existing services  Likely challenges to implementation |
| Indicators/measures of success | Ways of capturing whether/how the intervention was working |
