# Supplementary material for: Improving primary care based post-diagnostic support for people living with dementia and carers: Developing a complex intervention using the Theory of Change
Source: PLoS One. 2023 May 3;18(5):e0283818. doi: 10.1371/journal.pone.0283818 (PMC10155958; doi:10.1371/journal.pone.0283818)
Supplement: S3 File — (DOCX) [file pone.0283818.s003.docx]

## **Supplementary file 3: Illustrative case studies**

To illustrate how the PriDem intervention might affect the care of people living with dementia and carers, we outline common problems in existing post-diagnostic support and present fictional case studies to show the improvements anticipated.

Case study 1 concerns Mrs Forsyth who has recently been diagnosed with dementia. Common problems with current services at this point in the illness trajectory are:

- Most people will receive no active follow up by a memory assessment service
- No consistent system for allocating a named point of contact
- People and carers may feel abandoned
- People and carers receive little help with making sense of support services or seeking information appropriate to their needs.

| Case study 1: How the PriDem intervention will address these problems  Mrs Forsyth was diagnosed with vascular dementia at a memory assessment clinic. At a post-diagnostic visit with her daughter, Sheila, an information pack was provided, and they discussed driving and Lasting Power of Attorney (LPA) with the nurse. She was then discharged to her GP in primary care.  On receipt of the email from the memory clinic, her GP surgery asked Mrs Forsyth to make an appointment with the practice nurse. The practice nurse explored Mrs Forsyth’s understanding of the diagnosis and her feelings about it. She gave her a brief information sheet highlighting the areas in which support is available. Her information preferences were explored, and she was keen not to be overwhelmed with information, particularly about the later stages.  The practice nurse explained the role of the practice dementia team and that Mrs Forsyth will have a named point of contact to advise her on non-health related issues. Given that the situation was fairly settled, and that Mrs Forsyth seemed to be adjusting to the diagnosis, a social prescriber was allocated to be the named point of contact, and her details were provided. The practice nurse asked Mrs Forsyth to agree or consent to share details of her care with other health and perhaps any social care agencies, explaining that this can help avoid duplication and ensures that everyone has access to the same information. She also explored Sheila’s role, and with her permission recorded her contact details. As Sheila was registered with a different practice, the practice nurse suggested that she inform her own GP of her mother’s diagnosis.  The practice nurse also explained that everyone with dementia will have an annual review which is scheduled around the time of the patient’s birthday. As Mrs Forsyth’s birthday is not for another 10 months, she suggested that a review be scheduled in about four months to check how things are going. The practice nurse then summarised the meeting, noted the actions on a care plan and printed out a copy for Mrs Forsyth and Sheila. She checked whether Mrs Forsyth was happy to manage her own appointments, whether she would like to be sent a text reminder the day before any appointments, and whether she would also like the surgery to inform Sheila. She reassured her that these decisions can be revisited at any time.  A copy of the discharge letter, care plan and a brief summary of the diagnostic meeting were sent to the social prescriber who arranged an appointment with Mrs Forsyth in the next couple of weeks. Taking the lead from Mrs Forsyth, the discussion focused on practical issues, including help available with setting up an LPA. She explained that cognitive stimulation therapy (CST) has been shown to help people in the early stages of dementia and gave some examples of topics covered in the sessions. As Mrs Forsyth was interested, she provided the contact information for the local provider and explored practical issues relating to getting there. As the family was not keen on too much information at this stage, she did not suggest carer psychoeducation for Sheila, but made a note to consider this in future. She then summarised the actions, amended the care plan, and sent copies to Mrs Forsyth, Sheila and the GP surgery.  **Outcomes**   - Mrs Forsyth is now known to the practice dementia team - A named point of contact has been allocated and made an initial contact - Mrs Forsyth has received the initial help she needs and been signposted to relevant services. - Her care network is established, so that she knows who to contact for future advice - Her next review is scheduled. |
| --- |

Case study 2 concerns Mr Fields who has moderate dementia and his wife who is struggling to cope. Common problems with current services at this point in the illness trajectory are:

- Primary care professionals may not feel confident about managing emerging difficulties
- Primary care staff lack detailed knowledge of local services; this may result in multiple referrals to different agencies
- Agencies receiving referrals may be unclear what help is needed and each conducts their own assessment; some referrals may not be appropriate
- Community mental health teams may feel these problems are not severe enough to justify their involvement
- Primary care staff, people living with dementia and carers do not receive needed support
- Situations deteriorate until a crisis referral to secondary care is needed.

| Case study 2: How the PriDem intervention will address these problems  Mr Fields was diagnosed with vascular dementia three years ago and lives with his wife. They had been managing quite well, but his recent diabetes review raised concerns over whether he was taking his medication and managing his personal care. His annual dementia review was therefore brought forward by the practice nurse. The couple completed and returned the checklist of issues they wanted to discuss. When the practice dementia team looked over the list, they decided that it would be useful to have input from the practice nurse, social prescriber and community pharmacist. It was agreed that the community pharmacist would arrange a home visit; and, as she was newly appointed, the clinical dementia lead would accompany her to provide mentoring and support.  The review began with the couple talking to the social prescriber and practice nurse together. They started with an open question about how the couple were coping. While Mr Fields said that things were ‘champion’, they noticed Mrs Fields rolling her eyes as he spoke. Mrs Fields then described difficulties with managing her husband’s pills, his lack of interests outside the home, and problems with looking after himself properly. The practice nurse suggested that Mr Fields go with her to another room to get weighed, have his blood pressure and feet checked. This created opportunities for the couple to speak more frankly. Further exploration indicated that Mrs Fields was feeling isolated and stressed.  The social prescriber acknowledged the difficulties of Mrs Fields’ situation. She explained that she could refer Mrs Fields for a more detailed exploration of her needs by a carer support service which would identify services and support. Mrs Fields thought this would be helpful but commented that she would not be able to leave her husband and asked if a home visit would be possible. This enabled the social prescriber to mention services which could support her husband and give Mrs Fields a much-needed break. Mrs Fields did not think her husband would be interested but agreed that the social prescriber could discuss options with Mr Fields.  The other main issue for the couple related to personal care: Mr Fields was reluctant to shower or bathe. Although her husband did not admit it, Mrs Fields wondered whether this was linked to a couple of falls and near misses in the bathroom. Although he had not injured himself, he seemed less confident but was also very resistant to being helped. The social prescriber said that she would check that the practice nurse and community pharmacist were aware of the falls and reviewed any factors that could be causing the falls. She also suggested a referral to an occupational therapist who would visit them at home to discuss ways to make the bathroom safer to facilitate Mr Fields’ independence but noted that there was a waiting list for this service.  As Mrs Fields was also a patient at the practice, the social prescriber suggested that she should have a word with the practice nurse about her own health, while she talked to Mr Fields about possible social activities. The social prescriber and practice nurse swapped rooms with a quick chat to share key points from their separate discussions.  Now with Mrs Fields, the practice nurse explored any health concerns and checked whether she had lost weight and how she was sleeping. Given the number of issues raised, the practice nurse explored Mrs Fields’ priorities to avoid multiple referrals and assessments. They confirmed that Mrs Fields was happy to go ahead with both the carer assessment and occupational therapist visit.  Meanwhile the social prescriber had a chat with Mr Fields, exploring his interests and activities. He commented that he missed his allotment but had found it too much. As he was tired by this time, she suggested that she would look into some possible options and get back to him. Her main aim at this stage was just to raise the idea of new activities so she was keen to take things slowly.  Recognising that this is a challenging time, a review was scheduled for 3 months’ time to check that all the actions had been carried out and review the situation.  **Outcomes**   - Mrs Fields’ stress was acknowledged and explored - Priorities were agreed to avoid multiple referrals - Outstanding issues were recorded for follow up at the next appointment - Frequency of review was increased to check progress with actions - Referrals focused on specialist areas of need - The primary care team will seek advice on their management plan for Mr & Mrs Fields from the clinical dementia lead at the next multidisciplinary team meeting. |
| --- |
